# The Relative Influences of Government Funding and International Collaboration on Citation Impact

**DOI:** 10.1002/asi.24109

**Published:** 2018-11-22

**Authors:** Loet Leydesdorff, Lutz Bornmann, Caroline S. Wagner

**Affiliations:** ^1^ Amsterdam School of Communication Research (ASCoR), University of Amsterdam PO Box 15793, Amsterdam 1001 NG The Netherlands; ^2^ Division for Science and Innovation Studies Administrative Headquarters of the Max Planck Society Munich 80539 Germany; ^3^ John Glenn College of Public Affairs, Ohio State University Columbus OH 43210 USA

## Abstract

A recent publication in *Nature* reports that public R&D funding is only weakly correlated with the citation impact of a nation's articles as measured by the field‐weighted citation index (FWCI; defined by Scopus). On the basis of the supplementary data, we up‐scaled the design using Web of Science data for the decade 2003–2013 and OECD funding data for the corresponding decade assuming a 2‐year delay (2001‐2011). Using negative binomial regression analysis, we found very small coefficients, but the effects of international collaboration are positive and statistically significant, whereas the effects of government funding are negative, an order of magnitude smaller, and statistically nonsignificant (in two of three analyses). In other words, international collaboration improves the impact of research articles, whereas more government funding tends to have a small adverse effect when comparing OECD countries.

## Introduction

To view the national impact of international collaboration, Wagner and Jonkers (2017) assigned articles and impact measures to countries using fractional counting and a field‐weighted citation index (FWCI), as defined by the Scopus team at Elsevier (Plume & Kamalski, 2014). They found “a clear correlation between a nation's scientific influence and the links it fosters with foreign researchers.” (p. 32). The authors show that public R&D funding is only weakly correlated with the citation impact of a nation's articles. To reach this conclusion, the authors created an index of openness with values assigned for OECD countries. The data are available for download at go.nature.com/2fzrnt3.

The Comment in *Nature* remains at the level of pairwise correlations. In our opinion, these data allow for a next step: the effects of government funding and international collaborations on citation impact can be tested using regression analysis. Has international collaboration in the meantime become an independent factor in the self‐organization of the sciences (Persson, Glänzel, & Danell, 2004; Wagner & Leydesdorff, 2005; Wagner, Whetsell, Baas, & Jonkers, 2018)? Or is domestic stimulation by national governments a more crucial factor? It has been argued that the sciences are self‐organizing, and thus relatively resilient against changes in external funding priorities by governments (van den Daele & Weingart, 1975).

To test the hypothesis further, we scaled up to a decade of data (2003–2013) using the funding data (Government Budget Allocations for R&D; GBARD)^1^ of 35 OECD member states and seven affiliated economies,^2^ on the one side, and using our access to an in‐house version of the Web of Science (WoS) developed and maintained by the Max Planck Digital Library (MPDL, Munich), on the other. As in the study of Wagner and Jonkers (2017), we assume a delay of 2 years between funding and output and accordingly use OECD funding data for the period 2001–2011.^3^ Because we have a time‐series of observations, the publication year of the articles was added to the model as a third independent variable.

## Methods

FWCI is a relative measure, whereas our independent variables are numbers of articles and US$ normalized by the OECD as Purchasing Power Parity (PPP). In order to avoid problems with this difference in the scale of the measurement, we use percentile classes of articles as dependent variables at 50%, 10%, and 1% of the most frequently cited articles, normalized with reference to the corresponding subject categories in WoS and publication years (see Table 1). We added the number of papers for comparison. Only articles with the document type “article” are considered. In the case of ties in citation numbers at the respective thresholds, the countries' articles are fractionally assigned to the percentile classes (Waltman & Schreiber, 2013). The resulting numbers were rounded off.

**Table 1:** Key numbers for the variables included in the regression models.

Variables	Mean	Standard deviation	Minimum	Maximum
Articles	29,373.4	53,622.15	114	368,399
Top 1% articles	335.92	755.23	0	5,457
Top 10% articles	3,340.31	7,210.89	9	49,855
Top 50% articles	15,873.4	31,251.06	52	212,857
International collaboration	11,658.41	17,642.31	85	131,331
Expenditure (US Dollars, Millions)	8,390.895	21,975.7	24.66	164,292
Publication year	2,007.65	3.47	2,002	2,013

Three independent variables are used: (i) The annual number of internationally coauthored articles for each country; (ii) government budget allocations for R&D (GBARD) in the publication year *y* – 2 assuming expenditures to show output with a 2‐year lag; (iii) the publication year of the articles. In other words, changes over time are controlled while studying the relative influences of government funding and international collaborations on citation impact. Although standardized by the *Frascati Manual* (OECD, 1976 [1963]), however, the collection of input data by the OECD is decentralized in practice and therefore less reliable for comparisons among nations than WoS data (Aksnes, Sivertsen, van Leeuwen, & Wendt, 2017).

The dependent variables are count variables concerned with over‐dispersion, so we perform negative binomial regression models (Long & Freese, 2006). The regression models are based on *n* = 417 observations of “publication year x expenditure (country)” combinations. The countries are considered between 1 and 12 times in the analyses (on average, 11 times). The cluster option in Stata corrects the standard errors of the coefficients for the fact that we have more than 1 year for each country (Hilbe, 2014). We tested for multicollinearity of the independent variables, but found—according to the guidelines of Acock (2016)—scarcely any hint of a multicollinearity problem.

## Results

The results of the models show that the coefficients for international collaboration and expenditure are close to zero (see Table 2).

**Table 2:** Coefficients and *t* statistics from four negative binomial regression models.

	(1)	(2)	(3)	(4)
	Articles	Top 50% articles	Top 10% articles	Top 1% articles
International	0.00^***^	0.00^***^	0.00^***^	0.00^***^
collaboration	(4.82)	(4.98)	(5.13)	(5.30)
Expenditure	–0.00	–0.00	–0.00	–0.00**
(US Dollars, Millions)	(–0.41)	(–1.10)	(–1.95)	(–2.62)
Publication year	0.00	–0.01	–0.01	0.03*
	(0.11)	(–0.71)	(–0.43)	(2.25)
Constant	6.68	21.97	15.29	–51.49
	(0.32)	(1.12)	(0.72)	(–2.09)
Observations	417	417	417	417

*t* statistics in parentheses.

^*^ *p* < .05, ^**^ *p* < .01, ^***^ *p* < .001.

In order to interpret the results of the regression models, Table 3 shows average marginal effects. These effects are changes in the dependent variable when the independent variable is increased by one unit (and the other independent variables are set to the mean value).

**Table 3:** Marginal effects with one unit change in the independent variable (+1).

	Change	Confidence interval	
*Articles*			
International collaboration	1.253	0.874	1.631
Expenditure (US Dollars, Millions)	–0.167	–0.934	0.600
*top 50% articles*			
International collaboration	0.742	0.495	0.988
Expenditure (US Dollars, Millions)	–0.177	–0.471	0.117
*top 10% articles*			
International collaboration	0.161	0.104	0.218
Expenditure (US Dollars, Millions)	–0.049	–0.093	–0.004
*top 1% articles*			
International collaboration	0.016	0.010	0.022
Expenditure (US Dollars, Millions)	–0.005	–0.009	–0.002

The results can be interpreted as follows: on average, an increase of funding by one US$ million PPP decreases slightly the expected numbers in the 50%, 10%, and 1% most‐highly cited articles by 0.18, 0.05, and 0.01 articles, respectively. The decrease for the number of articles is 0.17. On average, the addition of one internationally coauthored article increases the expected numbers of articles in the 10% and 1% most‐highly cited articles by 0.2 and 0.02, respectively.

## Conclusion and Discussion

We confirm findings that international collaboration has a statistically significant and positive effect on the citation impact of nations. However, the effect is small. Government funding tends to have a negative or negligible effect on citation impact. However, our conclusions are “on average:” some nations appear to be more effective in turning funding into citation impact than others—several small nations punch above their weight in impact relative to spending (Sandström & Van den Besselaar, 2018).

Our results suggest diminishing returns of investments: additional government funding seems not to be absorbed by authors and institutions who produce more highly cited articles. It may well be that the influence of government funding for some (for instance, capital‐intensive) domains is different from others. Another factor behind the weak correlations may be the noted decentralization in the collection of input data by the OECD (Asknes et al., 2017). Leydesdorff and Wagner (2008) found large differences in the price (in US$) per article among nations. Some countries may have more slack and bureaucracy in the organization of the sciences than others (Taylor, 2016; cf. Shelton & Leydesdorff, 2012). These various possible explanations can be the subject of further research.

Policy towards R&D investment has been based on consensus that one needs more science to thrive in technology‐based growth (for instance, Coccia, 2010; Grupp, 1995). The underlying assumption has been that national agents are able to appropriate the benefits of national public spending. This research suggests that the links between funding and outputs are partly decoupled from a national base, especially at the international level (Wagner, 2008). This new configuration has implications for accounting for the benefits of public funding, which requires further research.

## Endnotes

1 We follow Wagner and Jonkers (2017) and use GBARD (OECD, 2017, p. 2; cf. Luwel, 2004, p. 327), and not Gross Expenditure in R&D (GERD) or Higher‐Education Expenditure in R&D (HERD). GERD includes business funding; with HERD, we would miss spending by government research institutes (including the academies), which are important producers of publicly funded scientific knowledge.

2 These seven countries are: Argentina, China, Romania, the Russian Federation, Singapore, South Africa, and Taiwan.

3 Funding data were retrieved from the OECD online at http://stats.oecd.org/Index.aspx?DataSetCode=MSTI_PUB .
